# Influence of quality of care and individual patient characteristics on quality of life and return to work in survivors of the acute respiratory distress syndrome: protocol for a prospective, observational, multi-centre patient cohort study (DACAPO)

**DOI:** 10.1186/s12913-015-1232-2

**Published:** 2015-12-17

**Authors:** Susanne Brandstetter, Frank Dodoo-Schittko, Sebastian Blecha, Philipp Sebök, Kathrin Thomann-Hackner, Michael Quintel, Steffen Weber-Carstens, Thomas Bein, Christian Apfelbacher

**Affiliations:** Medical Sociology, Institute of Epidemiology and Preventive Medicine, University of Regensburg, Dr.-Gessler-Str. 17, 93051 Regensburg, Germany; Department of Anaesthesiology, Operative Intensive Care, Regensburg University Hospital, Franz-Josef-Strauss-Allee 11, 93053 Regensburg, Germany; Department of Anaesthesiology and Intensive Care Medicine, Charité – University Medicine Berlin, Augustenburger-Platz 1, 13353 Berlin, Germany; Department of Anaesthesiology, Emergency and Intensive Care Medicine, University Medicine, Robert-Koch-Strasse 40, 37075 Göttingen, Germany

**Keywords:** Health-related quality of life, Return to work, QoC, ARDS, Study protocol

## Abstract

**Background:**

Health-related quality of life (HRQoL) and return to work are important outcomes in critical care medicine, reaching beyond mortality. Little is known on factors predictive of HRQoL and return to work in critical illness, including the acute respiratory distress syndrome (ARDS), and no evidence exists on the role of quality of care (QoC) for outcomes in survivors of ARDS. It is the aim of the DACAPO study (“Surviving ARDS: the influence of QoC and individual patient characteristics on quality of life”) to investigate the role of QoC and individual patient characteristics on quality of life and return to work.

**Methods/Design:**

A prospective, observational, multi-centre patient cohort study will be performed in Germany, using hospitals from the “ARDS Network Germany” as the main recruiting centres. It is envisaged to recruit 2400 patients into the DACAPO study and to analyse a study population of 1500 survivors. They will be followed up until 12 months after discharge from hospital. QoC will be assessed as process quality, structural quality and volume at the institutional level. The main outcomes (HRQoL and return to work) will be assessed by self-report questionnaires. Further data collection includes general medical and ARDS-related characteristics of patients as well as sociodemographic and psycho-social parameters. Multilevel hierarchical modelling will be performed to analyse the effects of QoC and individual patient characteristics on outcomes, taking the cluster structure of the data into account.

**Discussion:**

By obtaining comprehensive data at patient and hospital level using a prospective multi-centre design, the DACAPO-study is the first study investigating the influence of QoC on individual outcomes of ARDS survivors.

## Background

Health-related quality of life (HRQoL) and return to work are important outcomes in critical care medicine, reaching beyond mortality. Little is known on factors predictive of HRQoL and return to work in critical illness, including the acute respiratory distress syndrome (ARDS), and no evidence exists on the role of quality of care (QoC) for outcomes in survivors of ARDS.

The acute respiratory distress syndrome (ARDS) is a life threatening condition and is associated with significant morbidity and mortality up to 45 % [1]. ARDS is characterized by respiratory failure, caused either by direct pulmonary (e.g. pneumonia, aspiration) or indirect extra-pulmonary conditions (e.g. sepsis, massive transfusion) [2] and requires intensive medical care and implicit invasive mechanical ventilation.

During the last decades, much clinical research has been conducted on the management of patients with ARDS in intensive care units (ICUs). This body of research has focused primarily on the use of optimal ventilation strategies [3], pharmacological therapies [4] and the improvement of supportive strategies [5]. However, despite current treatment options in-hospital mortality in patients suffering from ARDS remains high [1, 6]. At the same time, the perspective on patients’ long-term functioning and HRQoL after ARDS has been growing in importance in recent years [7, 8]. Herridge et al. demonstrated that less than half of patients surviving ARDS could return to work within one year [9] and that survivors showed a decreased HRQoL and were substantially impaired in physical functioning even after 5 years [10].

Research on predictors of long-term outcomes in survivors of ARDS is quite scarce and the results are fragmentary. Up to now there is some evidence that higher social status and the availability of social support might be associated with better medium term health status [11, 12], whilst the increased prevalence of psychiatric symptoms represents a factor affecting HRQoL negatively [13]. One important aspect that is likely to determine outcomes in ARDS is the QoC that patients receive after developing the syndrome. In the different phases of the curative and rehabilitative treatment of ARDS the patients have to pass through various institutions of the health care system: i) intensive care treatment of the primary hospital referring the patient to a specialized ICU if the patient is not admitted directly to a highly specialized ICU, ii) inter-hospital transport from the referring to the specialized hospital, iii) intensive care treatment in the specialized hospital, iv) the rehabilitation unit. Scientific research investigating the impact of the health care provided in the different phases of ARDS treatment has not yet been conducted.

### Aim of the study and hypotheses

The DACAPO study aims at identifying predictive factors of HRQoL in survivors of ARDS in relation to QoC delivered in different phases and individual medical and psycho-social patient characteristics (“DACAPO: Surviving ARDS: the influence of quality of care and individual patient characteristics on quality of life”).

The primary hypothesis of the DACAPO study states that better QoC is associated with higher HRQoL and higher rates of return to work among patients who survive an ARDS. Secondary hypotheses are related to potential moderators between QoC and HRQoL: gender, socio-economic status, social support and psychopathological syndromes. Being female, having higher socio-economic status, the availability of higher levels of social support and not being affected by psychopathological syndromes should attenuate the associations between QoC, and HRQoL and return to work, respectively.

Additional research questions refer to the costs of the treatment of patients with ARDS and a long-term mortality follow-up.

## Methods

### Design

**Fig. 1:**
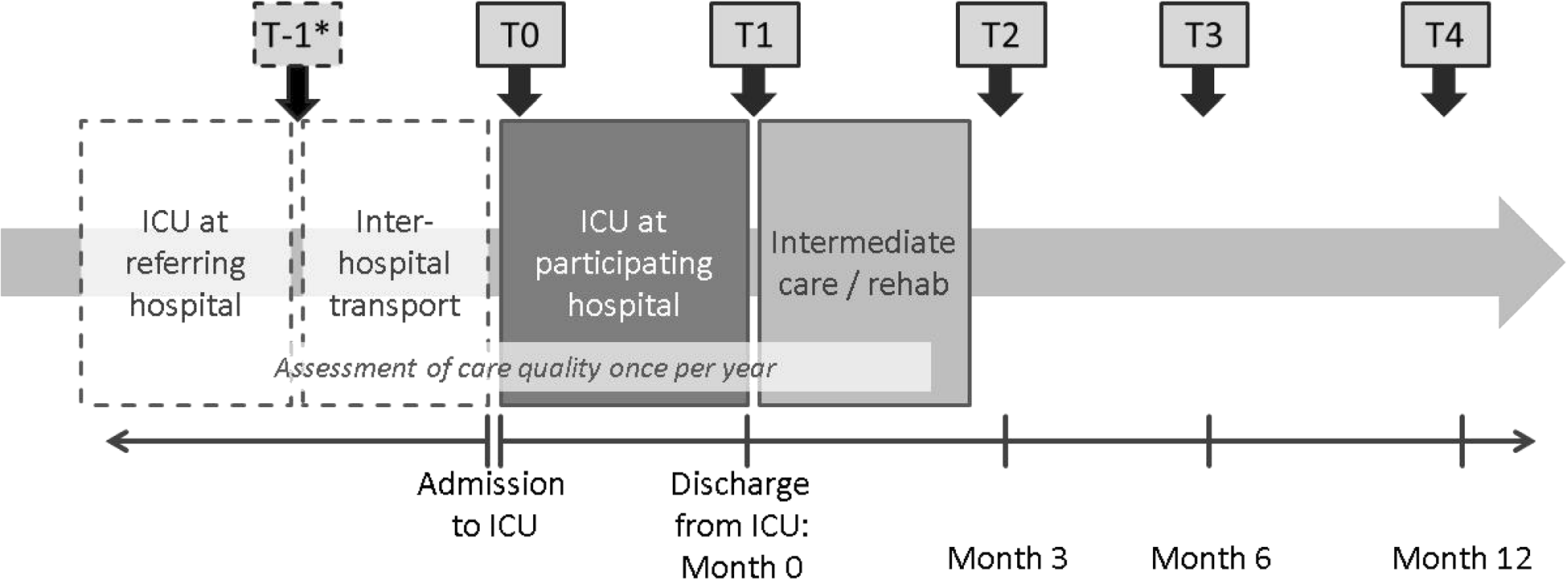
Study design. Note: * T-1 only applies to patients who have been treated in a referring hospital

DACAPO is an observational, prospective multi-centre patient cohort study. The study design is depicted in Fig. 1. Patients will be included in the study at the beginning of their ICU stay in a participating hospital (t0) and survivors will be followed over a period of 12 months after discharge from ICU. If patients have been treated before in a referring hospital, a retrospective assessment of their health status during their stay in this hospital and during inter-hospital transport will be performed. Four follow-up measurements (at discharge from ICU as well as 3, 6 and 12 months after discharge) will be performed on individual patient level.

### Sample & recruitment

#### Study sites

All 67 hospitals of the “ARDS Network Germany” (hospitals which are specialized in the treatment of patients with ARDS [14]) are invited to participate in the study. To ensure that the sample of participating hospitals is representative hospitals that do not belong to the ARDS network but provide care for patients with ARDS are also invited to participate.

#### Patients

The inclusion criteria for enrolment of patients are mechanical ventilation, PaO_2_/FiO_2_ (ratio of partial pressure arterial oxygen (PaO_2_) and fraction of inspired oxygen (Fi0_2_)) < 300 mmHg, and meeting the diagnostic criteria of the Berlin-definition [15]: i) Acute onset within one week, ii) bilateral pulmonary infiltrates (chest imaging), iii) respiratory failure not fully explained by cardiac failure or fluid overload. Further inclusion criteria are the patient being treated in any of the participating study sites and being 18 years old or older. Both female and male patients are being studied. In order to reflect the reality of health care delivery in ARDS, no exclusion criteria are applied.

### Sample size and patient flow

Studies on the incidence of ARDS report rates from 13 to 59 cases per 100.000 person-years [16]. Accordingly, for Germany the incidence could be estimated to be approximately 40.000 cases per year. Current data from the “ARDS Network Germany” lead to the assumption of approximately 40–50 cases per year per hospital.

**Fig. 2:**
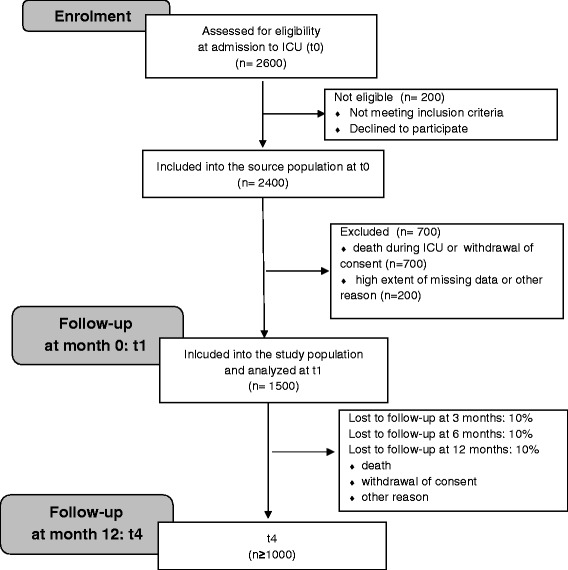
Expected patient flow

The estimated patient flow is depicted in Fig. 2. It is envisaged to screen 2600 patients for the presence of ARDS and to include 2400 patients as members of the source population (40–50 cases per year, with approx. 50 participating hospitals from the network resulting in 2000 to 2500 patients). Hospitals from outside the network will also contribute to the recruitment of patients. However, most of them are of modest or small size and they are not expected to include a substantial number of participants (5 to 10 patients each).

In-hospital mortality is assumed to be 30 %. Further, there will be some patients who will decline participation in the study, even if their carers had provided preliminary consent. Therefore, we expect to analyse a study population of 1500 patients at t1 (discharge from ICU). For each follow-up-assessment (t2-t4: 3 months, 6 months, 12 months after discharge), loss-to-follow-up due to death, withdrawal of consent or other reasons is estimated to be about 10 % [17], resulting in slightly more than 1000 patients at 12-months-follow-up.

### Measurements

Data will be captured at the *individual patient level* as well as *at the level of the health care institutions*.

The *main predictor* is QoC provided in the ICUs of the participating hospitals. In order to consider different domains of QoC the following main indicators were chosen: qualification of physicians (structural quality), implementation of routine daily multiprofessional ward rounds with documentation of daily therapy goals (process quality), number of ventilated patients per year (volume), and membership of the hospital in the “ARDS Network Germany” (general characteristics).

A wide range of additional indicators of QoC will be assessed for the ICUs of the participating hospitals, the ICUs of the referring hospitals, inter-hospital transportation, and rehabilitation units. Table 1 gives an overview on the different indicators organized by institution and domain. This information will allow for a comprehensive description of QoC in the different health care institutions which are involved in the care of patients with ARDS. Additional analyses will explore the associations between different indicators of QoC and patients’ outcomes in detail.

**Table 1:** Overview on data at the institutional level

	ICU of the referring hospital	Inter-hospital transport^a^	ICU of the participating hospital	Rehabilitation unit
*General characteristics*	ownership		ownership	ownership
	level of care		level of care	
	teaching status		teaching status	teaching status
	specialization of the ICU		specialization of the ICU	specialization
			**member of the ARDS network**	
		transportation via air or via ground		
*Structural quality*	qualification of direction, physicians and nurses	qualification of physicians and paramedics	**qualification of direction, physicians** and nurses	qualification of physicians, therapists and nurses
	24 h-availability of physicians and nurses		24 h-availability of physicians and nurses	24 h-availability of physicians and nurses
	physician/nurse-patient-ratio		physician/nurse-patient-ratio	physician/therapist/ nurse-patient-ratio
	availability of equipment for diagnostic and therapeutical procedures	availability of equipment for diagnostic and therapeutical procedures	availability of equipment for diagnostic and therapeutical procedures	availability of equipment for diagnostic and therapeutical procedures
*Process quality*	daily multiprofessional ward rounds with documentation of daily therapy goals	communication between physicians responsible for referral, transportation and admission	**daily multiprofessional ward rounds with documentation of daily therapy goals**	adoption of institutional and disesase-specific nursing and care concepts
	monitoring of sedation, analgesia and delirium		monitoring of sedation, analgesia and delirium	use of a nursing documentation system
	lung protective ventilation		lung protective ventilation	implementation of treatment pathways
	daily spontaneous breathing trials		daily spontaneous breathing trials	internal quality management
	documentation of structured caregiver-communication		documentation of structured caregiver-communication	documentation of patient-related therapy goals
	hand disinfection consumption		hand disinfection consumption	
	implementation of treatment pathways in accordance with up-to-date recommen-dations (e.g. early enteral nutrition, non-invasive ventilation)		implementation of treatment pathways in accordance with up-to-date recommen-dations (e.g. early enteral nutrition, non-invasive ventilation)	
*Volume*	number of patients per year		number of patients per year	number of patients per year
	number of ventilated patients per year		**number of ventilated patients per year**	number of ventilated patients per year
	number of patients died within 24 h per year		number of patients died within 24 h per year	

Notes: ^a^Data on inter-hospital transport is assessed at the individual patient level; however, it refers to the institutional level as it represents characteristics of quality of health care. **bold**: variables of main interest

As to date there are no agreed quality indicators for the treatment of patients suffering from ARDS, the selection of all quality indicators was informed by more generic recommendations regarding QoC of critically ill patients. We refer to the German quality indicators in intensive care medicine [18], the recommendations for inter-hospital transport by the German Interdisciplinary Society of Intensive Care Medicine (*Deutsche Interdisziplinäre Vereinigung für Intensiv- und Notfallmedizin*, DIVI) [19] and the recommendations regarding structural quality of rehabilitation centres made by the German Statutory Pension Insurance and the German Statutory Health Insurance [20].

All indicators will be assessed by questionnaire at the *institutional level.*

*Main outcome* measures of the DACAPO study are HRQoL and return to work among survivors of ARDS.

HRQoL will be assessed by the Short Form-12 self-report questionnaire (SF-12) [21]. It comprises twelve items which are scored according to published algorithms [22]. A physical and a mental component summary score can be derived, with higher values indicating higher HRQoL (normalized for the general population resulting in a standard score with mean = 50 and standard deviation = 10).

Return to work is operationalized by items representing two dimensions of survivors’ employment situation: i) date and ii) extent of return to work (same employment as before experiencing an ARDS, limitations due to ARDS-related disabilities, incapable of working).

*Moderating variables* are gender, socio-economic status (SES), prevalent and incident psychopathological symptoms and the availability of social support.

*Gender and SES* will be assessed as part of patients’ general characteristics. SES will be operationalized in two different ways: by the highest educational and professional level obtained by participants and by their subjective social status. The MacArthur-Scale assesses the subjective social status by asking people to place themselves on the scales of a ladder according to their perceived social position in their community [23].

The assessment of *psychopathological symptoms* focuses on affective disorders (major depression, panic disorder, post-traumatic stress disorder (PTSD)) and alcohol abuse. The validated German version [24, 25] of the Patient Health Questionnaire (PHQ) [26] is used to screen for the presence of a major depressive syndrome, a panic disorder syndrome, and an alcohol abuse syndrome. The instrument has been developed as a modular screening tool to establish provisional diagnoses according to DSM-IV (Diagnostic and Statistical Manual of Mental Disorders) [27]. The PTSS-14 (Post-Traumatic Stress Syndrome 14-Questions Inventory) [28] (German version: [29]) is employed to assess the prevalence of symptoms suggestive of PTSD, such as intrusions, avoidance behaviour or heightened irritability. The inventory has been adapted to traumata elicited by the experience of an ICU stay. Four items assess potential traumatic experiences during ICU in order to distinguish between ICU acquired and preexisting PTSD.

*Social support* will be assessed using the F-SozU K-14 [30]. The questionnaire covers three distinctive domains of social support (emotional support, instrumental support, social integration). The 14 items are scored on a 5-point Likert scale, with higher values indicating higher levels of social support.

In order to provide a *comprehensive description* of the study population and to account for *potential confounding variables*, additional information is assessed at the level of the individual patient. This comprises general medical ICU parameters (e.g. comorbidity, prognostic scores (SAPS (Simplified Acute Physiology Score) II, SAPS III [31, 32])), an organ dysfunction score (SOFA (Sequential Organ Failure Assessment score [33])), parameters relating to ARDS (e.g. cause and severity) and its treatment (e.g. ventilation parameters, use of supportive care measures, critical events (hypoglycaemia, hypoxia)) as well as socio-demographic and psycho-social characteristics. All parameters and their operationalization are listed in Table 2.

**Table 2:** Overview on data at the individual patient level: constructs and operationalizations

	**Constructs/concepts**	**Score/instrument**
*Socio-demographic characteristics*	age, **gender**	
	living situation, presence of informal caregiver(s), marital status	
	nationality	
	**educational and professional level**, employment	
	**subjective social status**	MacArthur-Scale [23]
*General medical characteristics*	height, weight, waist circumference	
	history of smoking, current smoking	
	comorbidity	according to SAPS III [32]
	history of psychiatric disorders	
*Medical characteristics relating to ARDS and its treatment*	cause and severity of ARDS	according to the “Berlin-Definition” [15]
	prognostic scores at ICU admission	SAPS II [31], SAPS III [32]
	organ dysfunction/failure	SOFA [33]
	blood gas analysis	
	ventilation parameters	
	presence of delirium	CAM-ICU, RASS [41]
	treatment with supportive care measures: ECMO, NO-inhalation, prone-positioning, muscle relaxant medication, tracheotomy	
	complications	
	length of ICU stay	
*Characteristics of health institution use*	length of hospital stay before referral to specialized centre, duration of inter-hospital transport, length and type of rehabilitative measures	
	outpatient health-service use	
*Psycho-social characteristics*	**prevalence of psychopathological syndromes**: major depressive syndrome, panic syndrome, PTSD, alcohol abuse	PHQ [26], PTSS-14 [28, 29]
	disability	Barthel-Index of Activities of Daily Living [42, 43]
	**social support**	F-SOZU [30]
	**healthrelated quality of life**	SF-12 [21]
	**return to work**	

Notes: **bold**: moderators and main outcomes

Additionally, costs will be assessed as direct costs in terms of treatment costs at the ICU.

The selection of measurements for the DACAPO study was driven by the scientific literature on HRQoL of survivors of ARDS and potential influential factors as well as by the expertise of collaborators from a wide range of disciplinary backgrounds.

### Data sources and data collection

A wide range of data sources and assessment methods will be used. Particular emphasis is being put on the use of routine data (e.g. data from patients’ health records) in order to minimize workload for the participating hospitals and to reduce the burden which additional diagnostic procedures and clinical evaluations might impose upon the patients and their carers.

Patients’ general medical characteristics and medical data relating to ARDS and its treatment will be assessed during ICU stay and gathered through *electronic case report forms* (eCRFs). Staff of the participating hospitals (physicians, study nurses) is being trained with regard to eCRF data entry. Most data will be extracted from ongoing documentations and patients’ health records, some data will have to be specifically assessed for the purpose of the study.

Data on the direct costs of treating patients with ARDS in the ICU will be gathered from hospital records as well. In Germany all hospitals are obliged to provide data on each patient’s medical treatments and procedures for the purpose of cost planning and statistics to the German Hospital Federation (German Hospital Fees Act, *Krankenhausentgeltgesetz* [34]). These data will be requested for the patients included in the study.

A 12-months mortality follow-up will be performed. Local municipal population registries will be contacted to obtain data on mortality at the patient level.

At baseline, caregivers will provide *proxy report* of patients’ socio-demographic data, at follow-ups (discharge from ICU, 3 months, 6 months, 12 months), patients themselves will complete *self-report* questionnaires on socio-demographic and psycho-social characteristics as well as on the main outcome measures. Questionnaires will be sent out by post. If they are not sent back within a period of four weeks, patients will be reminded by mail and phone. All attempts to contact patients will be documented.

Data on general hospital characteristics will be obtained from the generally accessible quality reports of German hospitals [35]. Data on indicators of QoC will be assessed through questionnaires administered to the directors of the hospitals/ICUs.

### Data management

With numerous hospitals participating in the DACAPO study, a web-based system for data entry seems to be most suitable. The open source software *OpenClinica*, version 3.1, will be used for electronic data collection and data management [36].

The IT center at the University of Regensburg will host the webserver for the *OpenClinica* instance. The web-based system has a secure communication protocol (https) with a digital certificate of SSL (=Secure Sockets Layer), as well as a reverse proxy as an additional security measure. The server will be updated frequently and backups will be performed daily.

eCRFs will be used for data entry. By enabling verification rules and edit checks, data entry persons will receive immediate feedback while the data are being entered into the system. In addition, queries can be deployed to validate missing or incomplete data or implausible values so that the supplied data remain consistent across all hospitals and high data quality is achieved. Furthermore, a continuous assessment of data quality will be performed by exporting data sets to statistical packages to run further error and plausibility checks.

Data from paper-pencil-questionnaires completed by patients will be entered into the database twice in order to ensure high data quality.

Personal data of participating patients will be strictly separated from the study data. Each participating patient will have an identification number for the study data and a separate identification number for his/her personal data. Both identifiers are pseudonymized. Only the trust office of the DACAPO study center is authorized to have access to the personal data of the enrolled patients. The data management is restricted solely to the study data. This separation is required in order to fulfill data protection guidelines.

### Statistical analysis

The analytical principles and statistical techniques to be employed in order to address the main aim of the DACAPO study (to identify the influence of QoC on survivors’ HRQoL and return to work) are as follows:

The selection of participating hospitals implies considerable baseline differences between the treated patients. To control for individual patient characteristics which might confound the relationship between QoC and the main outcomes, a wide range of variables are assessed. Using univariate analyses (*χ*^2^, T, Pearson’s r^2^), all of them will be checked for a potential confounding effect. Variables which are significantly related to predictor or outcomes in univariate analyses, will be included in the regression models.

The data resulting from the DACAPO study are organized in a hierarchical structure. While outcomes and potential confounders at the patient level (level 1) will vary between the patients, this does not apply to the predictor variables (QoC) at hospital level (level 2) which will be the same for all patients treated in a given hospital. This violates the statistical assumption of the independence of observations (i.e. measurements of patients within a hospital are more likely resembling each other than measurements of patients from different hospitals), resulting in an underestimation of standard errors by ordinary nonhierarchical regression models. Thus, multilevel modelling will be applied, a technique which allows to account for the grouping of patients into higher order clusters (hospitals). The modelling procedure will be carried out in three steps: i) A fully unconditional model *not* including level 1 and level 2 predictors will be fitted in order to determine the influence of the context (treatment in a given hospital) on outcome (HRQoL/return to work). The resulting intraclass correlation coefficient (ICC) describes the proportion of variance in the outcome variable explained by context. ii) Individual patient characteristics will be added to the model (fixed effects at level 1). iii) Context characteristics (QoC indicator of interest in each hospital) will be added to the model (fixed effects at level 2). The final model will allow for evaluating the effect sizes and the statistical significance of the influences of QoC and of individual patient-related variables on outcomes while considering the clustered structure of the data.

A linear multilevel regression model will be fitted both for the physical and the mental component summary score of the SF-12. A non-parametric multilevel regression model will be used for the outcome “return to work”.

The modelling procedure will be applied to each of the operationalizations of the predictor “QoC”. This approach will result in twelve final models (4 operationalizations of the predictor × 3 main outcomes). Thus, it will be crucial to consider the overall consistency of the associations as basis for the interpretation of the results and the decision about the hypothesis.

A power analysis has not been performed as all patients at participating study sites should be screened for eligibility.

All analyses will be carried out using SAS 9.4 software [37] and STATA version 12 software [38].

### Ethics and consent

The study has been approved by the ethic committee of the University of Regensburg (original approval: December 2013, approval of an amendment: June 2014; file number 13-101-0262) and by ethic committees of participating study centres. Due to the characteristics of ARDS and its treatment in the ICU (mechanical ventilation, analgosedation) the majority of patients will not be able to provide informed consent at the time of enrolment in the study. For the time period during which the patient is incapable of providing informed consent, patients’ caregivers or legal guardians will provide informed consent. Once the patient will be capable of providing informed consent he or she will be asked to confirm or disapprove the consent of the caregiver and to provide informed consent on his/her own.

### Governance

The study is jointly led by TB and CA (principal investigators) and has an independent advisory board composed of four experts from the fields of epidemiology, medical sociology, anaesthesiology and pneumology. Authorship for the research papers emerging from the DACAPO study will be assigned according to the recommendations of the International Committee of Medical Journal Editors [39]. Representatives of all centres that contribute patients to the study will be jointly named the “DACAPO study group” and will be acknowledged as such in each scientific paper emerging from the study.

## Discussion

The specification of an adequate study-design, an appropriate operationalization and a suitable statistical analysis with regard to the research questions of the DACAPO study, results in some inherent challenges arising from conceptual as well as from practical specifics:

It is assumed that efficacious and effective treatment and care of patients is facilitated by a high extent and quality of intersectoral collaboration between different health care institutions and health care professionals, whilst a lack of the former is made responsible for the provision of non-optimal treatment. This applies particularly to patients with long-lasting and complex conditions who pass through various health service institutions over the course of illness and recovery, as is expected to be the case for patients with ARDS. However, this fragmentation of the health care system and the assumed lack of collaboration do not only affect the treatment and care of patients but also the feasibility of research involving different health care institutions. Experiences made during the preparatory work for the DACAPO study suggest that professionals from the health care sectors responsible for in-hospital treatment and rehabilitation might not be well informed about their mutual practices regarding the care of patients with ARDS.

The DACAPO study aims to assess QoC within all health care institutions involved in the treatment of patients with ARDS. However, there is a lack of evidence-based quality measures which can be applied. Beyond, there are not even any established and consented guidelines on what good care of ARDS is and which specific interventions should be performed [5]. Thus, we use indicators of more general aspects of QoC (QoC in the ICU, QoC in rehabilitation units) which might not completely capture the specific features relevant for assessing the QoC provided for patients with ARDS.

Patients suffering from ARDS are study participants who are not capable of providing informed consent. Their caregivers need to consent to the participation in the study in lieu of the patients while they witness a life-threatening condition of a family member. This situation demands sympathetical and comprehensive information of patients’ caregivers provided through the medical professionals and may, nevertheless, result in lower participation rates. It is, therefore, particularly crucial to minimize loss-to-follow-up due to withdrawal of consent or failed attempts to contact participants. Otherwise, there might be concerns regarding the validity of the study results. A recent study described in detail the efforts made to gather follow-up measurements of patients surviving ARDS and strengthens the importance of repeated attempts to contact participants [40]. In order to assess attrition bias, we will perform a drop-out analysis, by comparing socio-demographic characteristics of those who drop out with those who will have complete follow-up data.

The output expected from the DACAPO study is as follows: By obtaining comprehensive data at patient and hospital level using a prospective multi-centre design, the DACAPO study will be the first study investigating the influence of QoC on individual outcomes of ARDS survivors. The envisaged sample size allows for complex analyses while considering potential moderating variables and confounders. No exclusion criteria will be applied. Thus, findings from this study are expected to have high external validity and therefore enable important insights into the real provision of health care services to patients suffering from ARDS in Germany. We expect to identify potential weaknesses in the treatment of patients with ARDS, particularly regarding the different involved health care institutions. Having uncovered these, further studies might target them and contribute to an improvement in the treatment of ARDS.

## Abbreviations

ARDS: acute respiratory distress syndrome
CAM-ICU: confusion assessment method for intensive care units
DSM-IV: diagnostic and statistical manual of mental disorders, 4^th^ edition
ECMO: extracorporeal membrane oxygenation
eCRF: electronic case report form
HRQoL: healthrelated quality of life
ICC: intraclass correlation coefficient
ICU: intensive care unit
PHQ: patient health questionnaire
PTSD: post-traumatic stress disorder
PTSS: post-traumatic stress syndrome 14-questions inventory
QoC: quality of care
RASS: Richmod Agitation and Sedation scale
SAPS: simplified acute physiology score
SES: socio-economic status
SF-12: short form-12 self-report questionnaire
SOFA: sequential organ failure assessment score
